# Carbon monoxide levels, smoking and adverse pregnancy outcomes

**DOI:** 10.1111/aogs.70068

**Published:** 2025-10-15

**Authors:** Katarzyna Galka, Michael Shea, Christina Y. L. Aye, Lawrence Impey

**Affiliations:** ^1^ Nuffield Department of Women's Reproductive Health, John Radcliffe Hospital Oxford University Oxford UK; ^2^ Fetal Medicine Unit, Women's Centre John Radcliffe Hospital Oxford UK

**Keywords:** birth weight, breath tests, carbon monoxide, cigarette smoking, pregnancy outcome, premature birth, small for gestational age

## Abstract

**Introduction:**

Identifying pregnant smokers is crucial for cessation support and increased fetal surveillance, but some patients may not disclose their smoking. Biochemical markers like breath carbon monoxide (CO) can improve detection, yet the optimal CO threshold for predicting smoking‐related risks remains unknown. Our objective was to assess the relationship between smoking, CO levels, and adverse pregnancy outcomes.

**Material and Methods:**

This retrospective cohort study analyzed 1 year of pregnancies (2023) in Oxford. Birthweight and adverse outcomes, small for gestational age (SGA), preterm birth (PTB), and extended perinatal mortality (EPM) were compared across CO categories and between self‐reported smokers and nonsmokers with CO ≤2 and >2 ppm. Statistical analyses included changepoint analysis, one‐way ANOVA, the Cochran–Armitage test for trend, binary logistic regression, and univariate linear regression.

**Results:**

Of 6963 pregnancies, 5041 (72.4%) had recorded CO levels. The mean gestation at birth was 39 + 6 weeks, and the mean birthweight (BW) was 3439 g (560); the mean BW centile was 54.25 (28.00). The prevalence of SGA, PTB, and EPM was 6.7%, 5.0%, and 0.6%, respectively. CO levels were >2 ppm in 11.7% of the cohort, including 5.0% of self‐reported non‐smokers. Changepoint analysis identified 2 ppm as the threshold for mean birthweight. Above this level, each 1 ppm increase in CO was associated with a 69.35 g decrease in BW and a 3.15‐point decrease in BW centile, indicating a dose–response relationship. Above 2 ppm, the odds of adverse outcomes were significantly increased: for SGA, OR 2.05–3.37; for PTB, OR 1.50–3.21; and for EPM, OR 2.52–4.22. Compared to non‐smokers with low CO, smokers with high CO had the highest risk of all outcomes. Non‐smokers with high CO had increased risks, but not significantly.

**Conclusions:**

At a threshold of >2 ppm, CO was associated with lower mean birthweight and higher rates of SGA and PTB, with a dose–response relationship. Universal CO testing could help identify and quantify risk in pregnancy.

## Graphical abstract

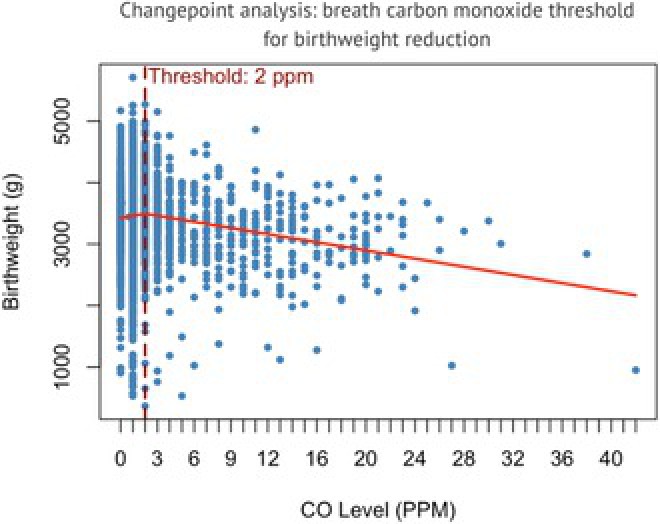

**Abbreviations**
BW: birth weight
CI: confidence interval
CO: carbon monoxide
EDD: estimated due date
EPM: extended perinatal mortality
GA: gestational age
OR: odds ratio
ppm: parts per million
PTB: preterm birth
SGA: small for gestational age

**Key message**

Breath carbon monoxide predicts low birthweight and adverse neonatal outcomes, with a clear dose–response above a threshold of >2 ppm. Implementing universal, repeated CO testing could help to identify pregnancies at risk.

## 1 INTRODUCTION

Smoking during pregnancy is a well‐documented public health concern. In England, despite record‐low smoking rates,^1^ 7.4% of women were smoking at the end of their pregnancy in 2023–24.^2^ It is associated with low birth weight and preterm birth^3^, ^4^ and is a leading modifiable risk factor for stillbirth. Quitting smoking significantly reduces these risks.^5^ It is therefore essential to identify pregnant smokers in order to provide appropriate cessation advice and increase fetal surveillance. However, not all women disclose smoking or smoking intensity, with non‐disclosure ranging from 3% to 75%.^6^, ^7^, ^8^, ^9^, ^10^

To address this, biochemical markers have been recently used. Breath carbon monoxide (CO) testing is a quick, cost‐effective, and noninvasive method.^11^, ^12^ In the United Kingdom, this test is now offered to all pregnant women at booking and at 36 weeks.^13^ Previous studies have examined the optimal CO threshold for identifying smokers and suggested different values, such as 3 ppm and 5 ppm. They found that birthweight was lower, and the risk of adverse pregnancy outcomes was higher in those with CO levels indicative of smoking.^14^, ^15^, ^16^, ^17^ However, the optimal CO threshold for predicting smoking‐related adverse outcomes remains unknown, including whether there is a dose–response relationship between CO levels and outcomes. It is also unclear if CO is a more reliable predictor of adverse outcomes than self‐reported smoking status.

This study aimed to identify the CO threshold and relationship with adverse neonatal outcomes and compare the use of CO with self‐reported smoking as risk indicators.

## 2 MATERIAL AND METHODS

### 2.1 Study design, setting, and population

This was a retrospective cohort study of 1 year of pregnancies (2023) using the OxGRIP dataset, which has been described previously.^18^ The dataset comprises routinely collected data on women receiving antenatal care and giving birth at Oxford University Hospitals. Pregnancies from 2023 were selected for analysis because CO data were only available for that year. Women with singleton, non‐anomalous pregnancies were included; exclusion criteria comprised multiple pregnancies, any fetal congenital abnormalities, pregnancies ending before 22 weeks, and those without CO measurements.

Ethical approval was granted by the South‐Central Hampshire Research Ethics Committee (reference 17/SC/0374) on July 26, 2017.

A woman was classified as a smoker if she self‐reported smoking at the booking appointment (median gestation 10 weeks) or any time during pregnancy. Carbon monoxide levels were measured at the booking visit using the Bedfont piCO™ Smokerlyzer® breath CO monitor (Bedfont Scientific Ltd., UK) and only ad hoc at other gestations: the highest CO level at any test was used.

### 2.2 Outcomes

Outcome measures were birthweight (BW) and BW centile, small for gestational age (SGA: <10th c^19^), preterm birth (PTB: 22 to <37 weeks), and extended perinatal mortality (EPM, intrauterine death or neonatal death within 28 days of birth). These outcomes were examined across groups stratified by CO thresholds.

We also compared outcomes between smokers versus nonsmokers and CO ≤2 ppm versus CO >2 ppm. We chose this threshold after changepoint analysis demonstrated that this level was associated with a significant reduction in mean BW. To examine unreported smoking or exposure, we stratified outcomes by both smoking status and CO levels: nonsmokers with CO ≤2 ppm, nonsmokers with CO >2 ppm, smokers with CO ≤2 ppm, and smokers with CO >2 ppm.

### 2.3 Statistical analysis

Most statistical analyses were conducted using IBM SPSS Statistics version 29.0.2.0. The chi‐squared test for trend (Cochran–Armitage test) and changepoint analysis were performed in R version 4.2.2. Demographic data were presented as mean (SD) for normally distributed variables, as median (interquartile range) for non‐normally distributed variables, and as n (%) for categorical variables.

Changepoint analysis was used to identify the CO exposure threshold associated with changes in birthweight. It was implemented using a segmented regression model via the *‘segmented’* package in R.^20^ Birthweight was chosen as the potentially most sensitive outcome to CO because it is a function of both growth and preterm birth. A one‐way analysis of variance (ANOVA) was used to compare mean birth weight across different approximately equal‐sized CO exposure categories and self‐reported smoking groups.

To assess the association between CO exposure and SGA, PTB, and EPM, a chi‐squared test for trend (Cochran–Armitage test) was conducted. Binary logistic regression was used to examine the association between the CO exposure categories and smoking with SGA, PTB, and EPM, analyzing smoking and CO exposure both combined and as separate predictors. The level of significance was set at *p* < 0.05 for all analyses, and confidence intervals were calculated at the 95% level.

To identify potential bias, we performed the following analyses. To assess whether CO data were missing at random, analyses were repeated on all deliveries with an EDD in 2023, including those with and without recorded CO levels. To determine whether the number of CO tests performed affected our results, we 1) examined the mean CO level according to the number of CO tests completed and 2) used linear regression to assess whether that relationship was independent of self‐reported smoking status. 3) We also performed a chi‐squared test for trend to assess whether self‐reported smokers were more likely than non‐smokers to undergo repeat CO testing.

## 3 RESULTS

**FIGURE 1:**
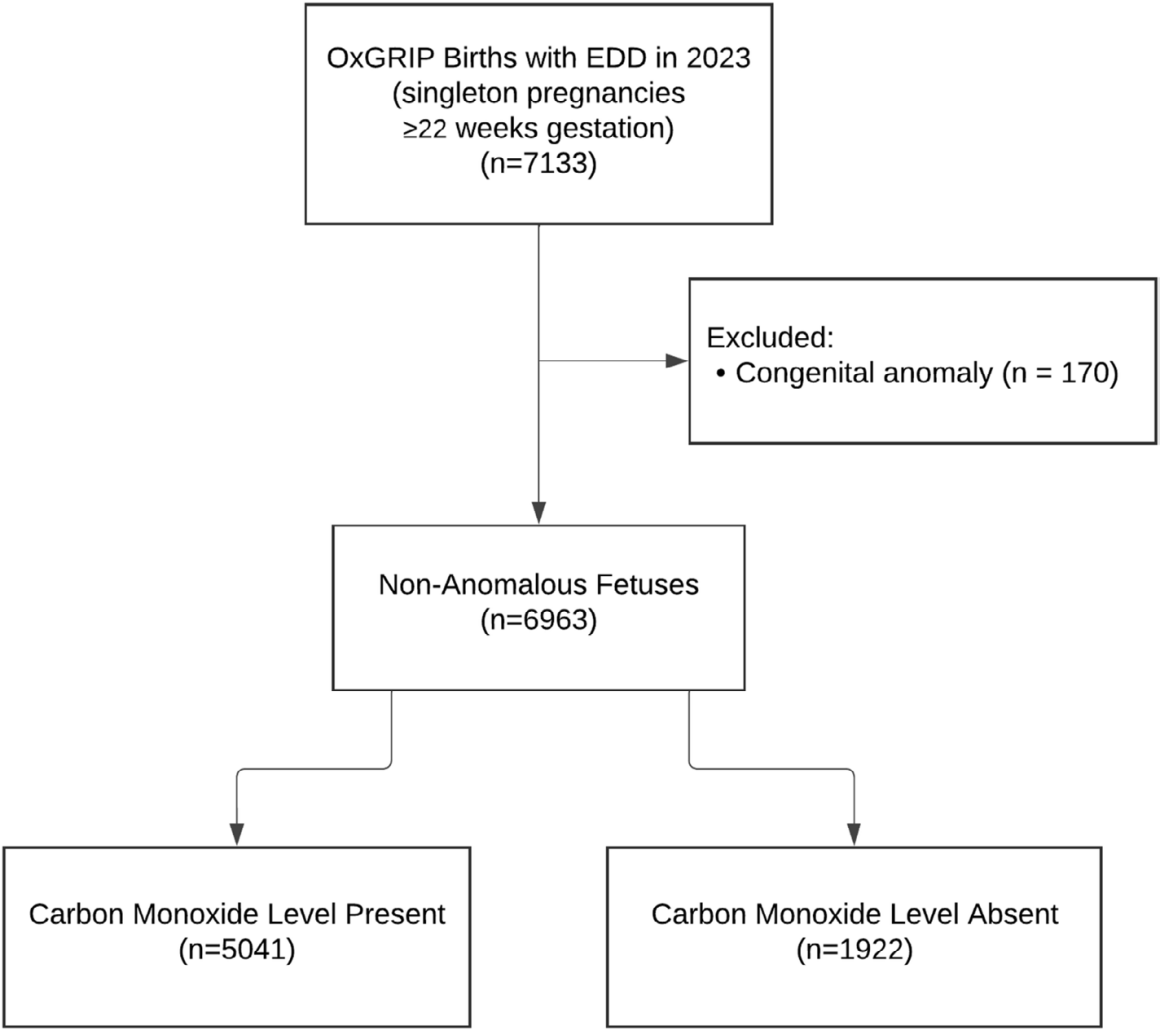
Flowchart summarizing population selection.

Of 6963 eligible pregnancies, 5041 (72.4%) women had CO levels recorded and were included in the final analysis (Figure 1).

Demographic characteristics are shown in Table 1. The mean birthweight of the cohort was 3439 (560); the mean birthweight centile was 54.25 (28.00). The prevalence of SGA, PTB, and EPM was 6.7%, 5.0%, and 0.6%, respectively. The incidence of self‐reported smoking was 9.2%, and CO levels were >2 ppm in 11.7%; 5.0% of the cohort were self‐identified nonsmokers with CO levels above 2 ppm. The majority of women (71.8%) had only one CO test during pregnancy, 25.9% had two tests, and just 2.2% had more than two.

**TABLE 1:** Demographic characteristics of study participants (*n* = 5041).

Characteristics	Value
Maternal age (years) mean (SD)	31.4 (5.3)
≥40	266 (5.3%)
Parity	
Nulliparous	2470 (49%)
Parous	2571 (51%)
Smoking in pregnancy	
Yes	466 (9.2%)
No	4575 (90.8%)
CO level	
≤2	4449 (88.3%)
3	184 (3.7%)
4–5	126 (2.5%)
6–10	129 (2.6%)
>10	153 (3.0%)
Ethnicity	
White	3853 (76.4%)
Asian	535 (8.8%)
Black	195 (3.9%)
Mixed	122 (2.4%)
Other	74 (1.5%)
Missing	262 (5.2%)
Index of multiple deprivation decile	
1–2 (most)	245 (4.9%)
3–4	428 (8.5%)
5–6	751 (14.9%)
7–8	1241 (24.6%)
9–10 (least)	1993 (39.5%)
Missing	383 (7.6%)
Body mass index	
Underweight (<18.5 kg/m^2^)	156 (3.1%)
Normal weight (18.5–24.9 kg/m^2^)	2465 (48.9%)
Overweight (25.0–29.9 kg/m^2^)	1406 (27.9%)
Class‐1 obesity (30.0–34.9 kg/m^2^)	622 (12.3%)
Class‐2–3 obesity (≥35.0 kg/m^2^)	386 (7.7%)
Missing	6 (0.1%)
Neonatal sex	
Female	2439 (48.4%)
Male	2602 (51.6%)
GA at delivery (weeks)	39 + 6 (1 + 6)
Birth weight (g) mean (SD)	3439 (560)
Birth weight (centile), mean (SD)	54.25 (28.00)
SGA (<10th centile)	338 (6.7%)
PTB (<37 + 0 weeks)	251 (5.0%)
EPM (intrauterine death or neonatal death within 28 days of birth)	28 (0.6%)

*Note*: Data are given as *n* (%) or mean (SD).

Abbreviations: BW, birthweight; CI, confidence interval; CO, carbon monoxide; EPM, extended perinatal mortality; GA, gestational age; OR, odds ratio; PTB, preterm birth; SGA, small for gestational age.

### 3.1 Carbon monoxide threshold for predicting birthweight

**FIGURE 2:**
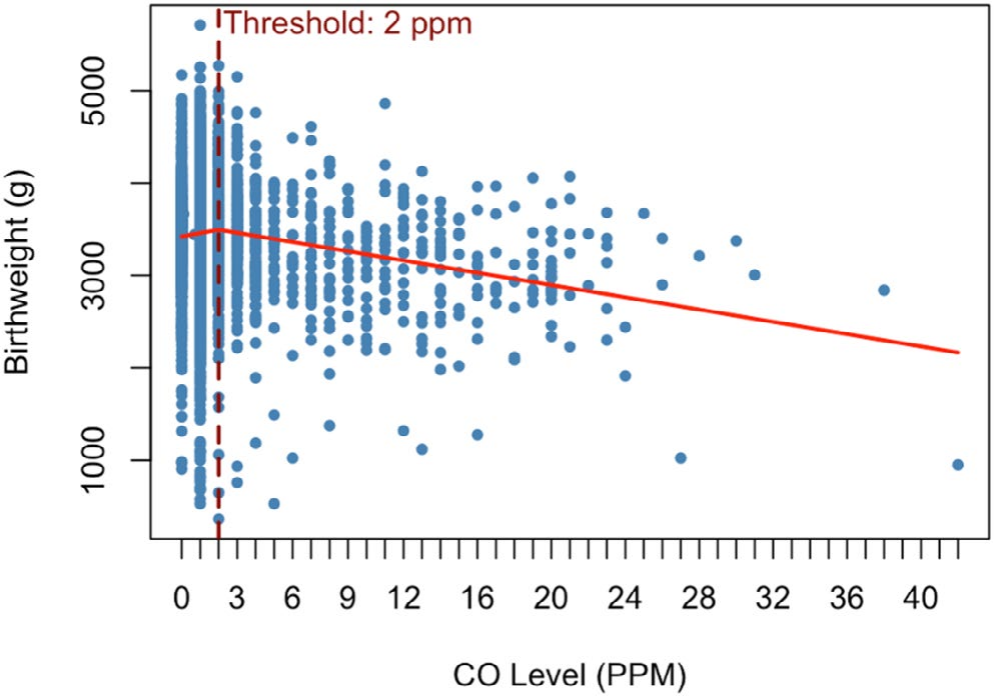
Changepoint analysis of breath carbon monoxide (CO) level and birthweight.

Figure 2 shows the results of the changepoint analysis, illustrating the estimated CO exposure threshold of 2 ppm. Based on the identified threshold, CO levels >2 were further categorized into approximately equal groups for subsequent analyses.

### 3.2 Dose–response relationship and adverse outcomes according to CO level

As a continuous variable, CO level was significantly associated with both birthweight and birthweight centile. Above the threshold of 2 ppm, each 1 ppm increase in CO corresponded to a 69.35 g decrease in birthweight (SE = 14.53, *p* < 0.001) and a 3.15‐point decrease in birthweight centile (SE = 0.73, *p* < 0.001). The chi‐squared test for trend showed a significant association between higher CO levels and increased risk of both SGA (*p* < 0.001) and PTB (*p* = 0.001), also indicating a dose–response relationship. A dose–response relationship with EPM was unproven given small numbers.

Table 2 shows mean birthweight, mean birthweight centile, and neonatal outcomes stratified by CO levels. The increasing risks of adverse outcomes with higher CO levels are evident. Above 2 ppm, the OR of all adverse outcomes is significantly increased. At very high CO levels (>10 ppm), the OR of EPM is 4.22 (1.24–14.29), of PTB is 3.21 (1.99–5.19), and of SGA is 2.75 (1.74–4.36).

**TABLE 2:** Mean birthweight and adverse outcomes across CO exposure groups.

	CO ≤2 *n* = 4449	CO >2 *n* = 592	*p*‐value	CO 3 *n* = 184	CO 4–5 *n* = 126	CO 6–10 *n* = 129	CO >10 *n* = 153	*p*‐value
BW (g), mean (SD) (95% CI)	3461 (548) (3445–3477)	3275 (615) (3225–3324)	<0.001	3510 (552) (3430–3590)	3297 (594) (3192–3402)	3172 (587) (3070–3275)	3060 (633) (2959–3161)	<0.001
BW (centile), mean (SD) (95% CI)	55 (28) (54–56)	48 (29) (46–50)	<0.001	59 (27) (55–63)	48 (28) (43–53)	42 (29) (37–47)	41 (27) (37–45)	<0.001
SGA *n* (%)	269 (6.0%)	69 (11.7%)	<0.001	8 (4.3%)	15 (11.9%)	23 (17.8%)	23 (15.0%)	<0.001
SGA OR (95% CI)	1.00 (ref)	2.05 (1.55–2.71)	<0.001	0.71 (0.34–1.45)	2.10 (1.21–3.65)	3.37 (2.11–5.38)	2.75 (1.74–4.36)	<0.001
PTB *n* (%)	210 (4.7%)	41 (6.9%)	0.020	6 (3.3%)	7 (5.6%)	7 (5.4%)	21 (13.7%)	<0.001
PTB OR (95% CI)	1.00 (ref)	1.50 (1.06–2.12)	0.021	0.68 (0.30–1.55)	1.19 (0.55–2.58)	1.16 (0.53–2.51)	3.21 (1.99–5.19)	0.001
EPM *n* (%)	21 (0.5%)	7 (1.2%)	0.029	2 (1.1%)	1 (0.8%)	1 (0.8%)	3 (2.0%)	0.122
EPM OR (95% CI)	1.00 (ref)	2.52 (1.07–5.96)	0.035	2.32 (0.54–9.96)	1.69 (0.23–12.64)	1.65 (0.22–12.34)	4.22 (1.24–14.29)	0.017

Abbreviations: BW, birthweight; CI, confidence interval; CO, carbon monoxide; EPM, extended perinatal mortality; GA, gestational age; OR, odds ratio; PTB, preterm birth; SGA, small for gestational age.

### 3.3 Smoking status versus CO level as predictors

Table 3 presents mean birthweight, mean birthweight centile, and neonatal outcomes stratified according to self‐reported smoking status and CO level >2. Compared with non‐smokers who had low CO levels, smokers with high CO levels had an increased risk of all outcomes. The risk of adverse outcomes in self‐reported nonsmokers with a high CO level was increased but not significantly. The risk of preterm birth remained in smokers even if they had a CO level ≤2; the risk of other adverse outcomes was increased but not significantly.

**TABLE 3:** Mean birthweight and neonatal outcomes by combined smoking status and CO level.

	Nonsmoker CO ≤2 *N* = 4324	Nonsmoker CO >2 *N* = 251	Smoker CO ≤2 *N* = 125	Smoker CO >2 *N* = 341	*p*‐value
BW (g), mean (SD) (95% CI)	3464 (547) (3448–3481)	3460 (578) (3388–3532)	3345 (590) (3241–3450)	3138 (606) (3074–3203)	<0.001
BW (centile), mean (SD) (95% CI)	55 (28) (54–56)	56 (28) (52–59)	51 (28) (46–56)	42 (28) (40–45)	<0.001
SGA *n* (%)	257 (5.9%)	21 (8.4%)	12 (9.6%)	48 (14.1%)	<0.001
SGA OR (95% CI)	1.00	1.45 (0.91–2.30)	1.68 (0.92–3.09)	2.59 (1.86–3.61)	<0.001
PTB *n* (%)	193 (4.5%)	9 (3.6%)	17 (13.6%)	32 (9.4%)	<0.001
PTB OR (95% CI)	1.00	0.80 (0.40–1.57)	3.37 (1.98–5.73)	2.22 (1.50–3.28)	<0.001
EPM *n* (%)	20 (0.5%)	2 (0.8%)	1 (0.8%)	5 (1.5%)	0.101
EPM OR (95% CI)	1.00	1.73 (0.40–7.44)	1.74 (0.23–13.03)	3.20 (1.19–8.59)	0.014
Median CO (ppm)	1.00	3.00	1.00	9.00	<0.001

Abbreviations: BW, birthweight; CI, confidence interval; CO, carbon monoxide; EPM, extended perinatal mortality; GA, gestational age; OR, odds ratio; PTB, preterm birth; SGA, small for gestational age.

To investigate this apparent importance of smoking even in women with a CO level ≤2, we investigated the relationship between the number of tests and both the CO level and smoking. Table 4 presents the number of CO tests completed and the corresponding mean CO levels.

**TABLE 4:** Number of CO tests and mean CO levels.

Number of CO tests	*n*	% of cohort	CO ppm mean (SD) (95% CI)
1	3619	71.8%	1.57 (2.84) (1.48–1.66)
2	1304	25.9%	1.83 (4.18) (1.60–2.05)
3	108	2.1%	2.06 (2.54) (1.57–2.54)
4	8	0.2%	5.19 (4.40) (1.51–8.86)
5	2	0.04%	15.00 (4.24) (−23.12 to 53.12)

Abbreviation: CO, carbon monoxide.

A one‐way ANOVA indicated that the mean CO level differed significantly according to the number of tests completed, *F*(4, 5036) = 12.80, *p* < 0.001. Linear regression showed that the association between the number of CO tests and mean CO level remained statistically significant after adjusting for smoking (*p* = 0.005), but the effect size was small, with mean CO increasing by only 0.2 ppm for each additional test. A chi‐squared test for trend showed no significant difference in the proportion of women with more than one CO test between smokers and nonsmokers, *χ* ^2^(1) = 1.84, *p* = 0.175. The comparison of CO levels between self‐reported smokers and nonsmokers is presented in Figure S1.

### 3.4 Missing carbon monoxide data

To assess whether missing CO data were missing at random, we compared pregnancy outcomes between those with and without CO measurements (Table S1). Women who reported smoking during pregnancy were more likely to undergo CO testing than nonsmokers (76.5% vs. 72.0%, OR 1.27; 95% CI: 1.042–1.544, *p* = 0.018).

## 4 DISCUSSION

In this study, we show that, at a threshold of >2 ppm, CO was associated with lower mean birthweight. A dose–response relationship exists, and increasing CO level is associated with lower birthweight centile and higher rates of SGA and PTB.

Smokers with a CO level ≤2 had a significantly higher risk of preterm birth, although the associations with size were not significant. It seems likely, given that the more frequently CO was measured, the higher the level, that had more measurements been done in each pregnancy the association would disappear. This is more biologically plausible than smoking being an independent risk factor and suggests that CO testing should be performed more than once: Infrequent CO monitoring may be inadequate to detect clinically significant smoking.

Surprisingly, nonsmokers with a CO >2 ppm did not have a higher risk of adverse outcomes. Reynolds et al.^16^ found that “non‐disclosers” (CO ≥3 but self‐reported nonsmokers), who comprised 43% of women with levels ≥3, had lower birthweights than nonsmokers with a low CO level. Our rate of CO >2 in nonsmokers was 5.0%. That using CO >2 does not apparently “detect” possible smokers is likely to be because this group had much lower CO levels (3 ppm) than smokers (9 ppm): A level of 2 should still probably be considered abnormal.

The two earliest studies that examined CO and smoking used an arbitrary 5 ppm CO cutoff.^14^, ^15^ Gómez et al. found a significant reduction in birthweight above this threshold.^15^ Secker‐Walker et al. observed the same relationship but only when CO was measured at the first visit; at 36 weeks, a significant reduction in birthweight was seen only at CO levels 10 ppm.^14^ Reynolds et al.^16^ suggested a threshold of 3 ppm but only 53 women had this level, and comparison was with 3 ppm and above. Our larger sample suggests that >2 is a more accurate threshold, corresponding to 11.7% of pregnancies. However, our data also indicate that more frequent testing might reveal an even higher percentage. However, the actual CO level, rather than a threshold, is most important. The cutoff for increased surveillance could vary according to resources, but the actual level should be used when risk is modeled.

Three studies found that mean birthweight decreased in a dose‐dependent manner with higher CO levels.^14^, ^15^, ^16^ Secker‐Walker et al. reported that CO levels measured at 36 weeks of gestation had a stronger negative association with birthweight than those recorded at the first prenatal visit.^14^ In contrast, Reynolds et al. showed that this relationship was similar at both time points. Gómez et al. found that mean birthweight in the 6–10 ppm group was 450 g lower than in the 0–5 ppm group, with this trend continuing at higher exposure levels (11–20 ppm and >20 ppm).^16^ We observed this effect across narrower CO ranges, demonstrating that each 1 ppm increase in CO was associated with a reduction in mean birthweight and showed this dose response extends to SGA and PTB.

The main limitations of this study include its retrospective design and the relatively small number of adverse pregnancy outcomes. We also did not have information on the number of cigarettes smoked, although this latter measure is likely to be imprecise. Although likely, it remains uncertain whether more frequent CO testing would overcome the apparent independence of smoking. Finally, CO data were not missing at random: Women who reported smoking were more likely to undergo CO testing. This limits comparison with smokers but does not alter our findings regarding the relationship between CO levels and adverse outcomes.

## 5 CONCLUSION

Breath carbon monoxide predicts low birthweight and adverse neonatal outcomes with a clear dose response above a threshold of >2 ppm. Implementing universal CO testing could help to identify at‐risk pregnancies, and this would need to be performed several times.

## AUTHOR CONTRIBUTIONS

LI conceived the project idea. LI, CYLA, and KG planned the project. KG and MS conducted the data analysis. KG wrote the original manuscript, which was revised by MS, CYLA, and LI. The manuscript was approved by all authors before submission.

## CONFLICT OF INTEREST STATEMENT

The authors declare no conflicts of interest.

## ETHICS STATEMENT

Ethical approval was granted by the South‐Central Hampshire Research Ethics Committee (reference 17/SC/0374) on July 26, 2017.

## Supporting information


Data S1.

## Data Availability

The data that support the findings of this study are available from the corresponding author upon reasonable request.
