# Supplementary material for: Carbon monoxide levels, smoking and adverse pregnancy outcomes
Source: Acta Obstet Gynecol Scand. 2025 Oct 15;104(12):2237–43. doi: 10.1111/aogs.70068 (PMC12668819; doi:10.1111/aogs.70068)
Supplement: Supplementary file 1 — Data S1. [file AOGS-104-2237-s001.docx]

**Supplement:**

*Supporting Information Table S1: Assessment of Missing at Random*

| Outcome | CO Level Present (N=5041) | CO Level Missing (N=1922) | OR (95% CI) | p-value |
| --- | --- | --- | --- | --- |
| **Birth Weight (BW)**  **(mean ± SD, 95% CI)** | 3439.23 (± 559.71) (3423.78–3454.69) | 3368.21 (± 682.65) (3337.67–3398.74) | - | <0.001 |
| BW (centile), mean (SD) (95% CI) | 54.25 (±28.00) (53.47 to 55.02) | 54.11 (±28.24) (52.84 to 55.37) | - | 0.857 |
| **Small for Gestational Age**  **(SGA)** | 338 (6.7%) | 141 (7.4%) | 1.11 (0.90–1.36) | 0.32 |
| **Preterm Birth (PTB)** | 251 (5.0%) | 153 (8.0%) | 1.65 (1.34–2.03) | <0.001 |
| Extended perinatal mortality (EPM) | 28 (0.6%) | 18 (0.9%) | 1.69 (0.93–3.07) | 0.08 |

Abbreviations: BW, birthweight, CO, carbon monoxide, GA, gestational age, SGA, small for gestational age, PTB, EPM, extended perinatal mortality, OR, odds ratio

*Supporting Information Table S2:* Birthweight versus CO level at the ‘threshold’ of 2 restricted to women who only had one measurement

|  | CO ≤ 2  n=3278 | CO> 2  n =341 |
| --- | --- | --- |
| BW (g), mean (SD) (95% CI) | 3437 (574) (3418 to 3457) | 3193 (620) (3126–3259) |


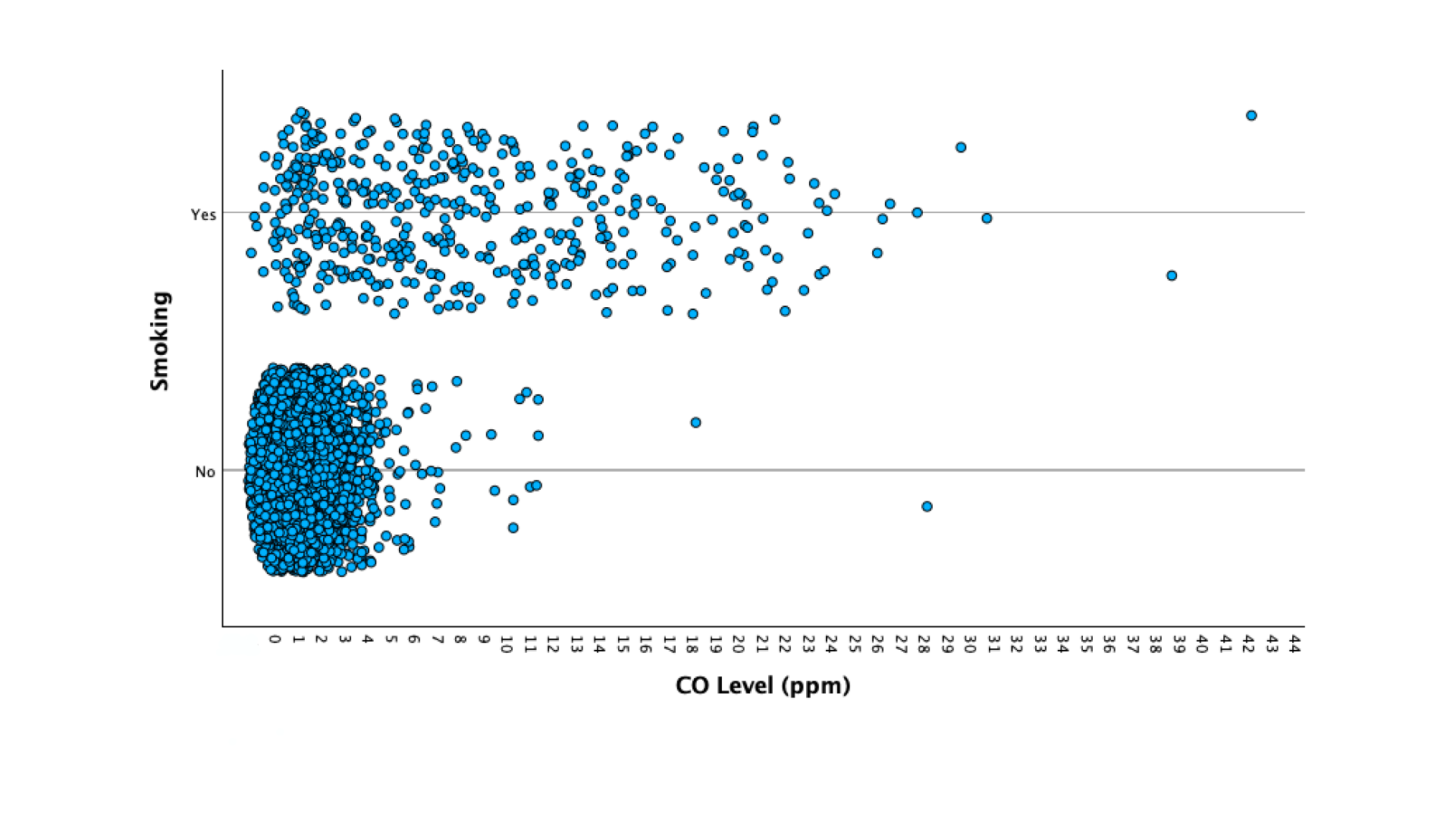
*Supporting Information Figure 1: Dot plot of CO level by reported smoking*
